# Unusual Surgical Resection of Asymptomatic Schwannoma of the Cervical Vagus Nerve With Risk of Stroke: Case Report

**DOI:** 10.1155/cris/9443139

**Published:** 2025-01-23

**Authors:** Roberto Sérgio Martins, Adilson J. M. de Oliveira, Evander Lucas, Mario Gilberto Siqueira

**Affiliations:** ^1^Peripheral Nerve Surgery Unit, Division of Neurosurgery, Hospital das Clínicas, Faculty of Medicine, São Paulo University, São Paulo, Brazil; ^2^Centro de Estudos Avançados em Formação e Educação Médica—CEDUMED, Faculty of Medicine, Agostinho Neto University, Luanda, Angola

**Keywords:** schwannoma, surgery, tumor, vagus nerve

## Abstract

Schwannomas are the most common tumors of the peripheral nerves, originating from their support cells, the Schwann cells. The location of the tumor in the vagus nerve is rare. Vagus schwannomas usually present as a solitary, slow-growing, asymptomatic mass that rarely causes neurological alterations. The differential diagnosis of vagus nerve schwannomas includes other tumors of the parapharyngeal space or neoplasms of the jugular foramen. We report the case of a patient with an asymptomatic schwannoma of the vagus nerve involving important neck structures, with radiological compression of the carotid artery with a high risk of stroke; because of this, we underwent surgery using a transcervical approach with intracapsular excision of the tumor. The patient has a good outcome. In asymptomatic patients' surgical indication is not an easy decision; in this case, the main reason for surgical indication was the risk of stroke with potential neurological sequels.

**Summary**

• A rare location of vagus nerve schwannoma is identified in an asymptomatic patient. Due to its relationship with important structures, a surgical approach is decided upon, leading to a favorable outcome.
• Asymptomatic cervical masses may represent lesions with potential for malignant transformation, making histological diagnosis important.
• Cervical schwannomas of the vagus nerve should be investigated with studies of the cervical vessels to rule out compression of the carotid and vertebral arteries.
• Surgery may be an effective option to prevent malignant transformation.

## 1. Introduction

Schwannomas are the most common tumors of the peripheral nerves, originating from their support cells, the Schwann cells [1]. They are relatively rare, accounting for approximately 5% of benign soft tissue tumors in adults [2]. The main sites of concentration are the cervical plexus and peroneal, ulnar, and vagus nerves [3]. Most lesions are solitary, asymptomatic, and characterized by slow growth [4]. Malignant transformation of tumors can be considerable, occurring in up to 13% of lesions [5]. Surgery is the definitive treatment for peripheral nerve schwannoma [6]. However, as it is an intrinsic tumor of the nerve, its removal poses a risk of new neurological deficits [7]. This fact, along with the observation that tumor growth is slow in approximately 60% of cases. Lubelski et al. [8] report this finding, which may support a more conservative approach in some situations.

If, in general, schwannomas are infrequent tumors, their occurrence in the cervical portion of the vagus nerve is even less common and, due to its rarity, a frequent subject of case reports [9]. Unlike neural sheath tumors of the extremities, vagus nerve schwannomas occur in a critical area in terms of important anatomical structures that are related to the lesion, such as the internal carotid artery [10]. On the one hand, this location leads to greater risks of direct injury to these important structures; on the other hand, there is a greater tendency to adopt surgical treatment even in minor and incidental lesions, reducing the morbidity of surgery.

In this context, we present the case of a patient with an incidental schwannoma of the cervical portion of the vagus nerve in order to discuss whether, due to the peculiarities of the location of this lesion, early surgery in asymptomatic cases is indicated.

## 2. Case History and Examination

**Figure 1:**
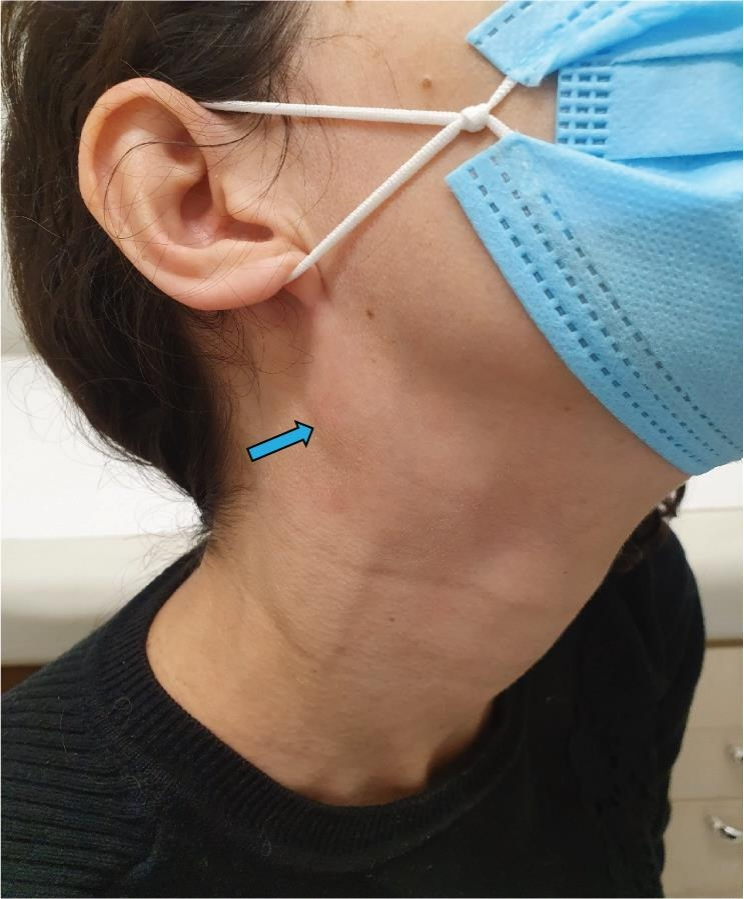
Representation case. Arrow—tumefaction upper cervical region suggesting a benign lesion.

A 38-year-old female patient who, during a cervical extension movement, noticed a right upper cervical nodule (Figure 1). There were no associated symptoms, including altered swallowing and phonation or any neurological complaints such as shock, burning, or numbness.

On examination, it was possible to identify an elliptical nodule with a fibroelastic consistency in the posterior triangle of the neck on the right side, approximately 4 cm in diameter, anterior to the sternocleidomastoid muscle, and extending to the angle of the mandible, which could be mobilized, with a negative Tinel's sign. Examination of the oropharynx showed no alterations, and assessment of the cranial nerves was normal. Dermatological lesions were not observed.

## 3. Investigation and Treatment

**Figure 2:**
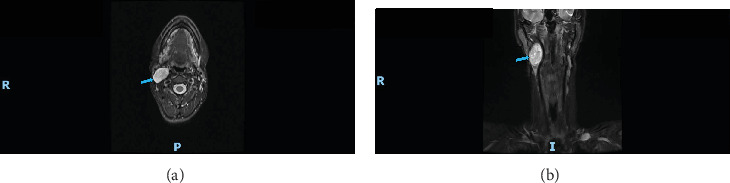
Angiographic MRI. (A) Axial view and (B) coronal view.

**Figure 3:**
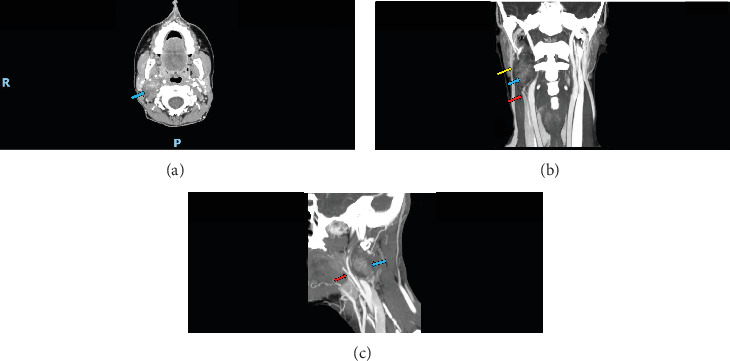
Angio CT scan. (A) Axial view, (B) coronal view, and (C) sagittal view. Blue arrow—tumor; Red arrow—carotid artery compression; Yellow arrow—vertebral artery compression.

Magnetic resonance imaging showed a fusiform, well-defined expansive lesion with predominantly defined limits in the right carotid space, measuring 4.5 cm × 3.0 cm × 1.7 cm in the longest axes. It showed increased signal on MRI T1 and T2 sequences (Figure 2), intense and progressive contrast uptake, especially in the central region, and no restriction to water diffusion. The lesion was located on the topography of the vagus nerve and displaced the internal carotid artery anteromedially and the internal jugular vein laterally, which was compressed by the lesion (Figure 3). It maintained contact with the medial pterygoid muscle, mandibular ramus, anterolateral margin of C2, deep margin of the parotid gland, paravertebral musculature, and the sternocleidomastoid muscle, with no signs of involvement of these structures (Figure 3).

**Figure 4:**
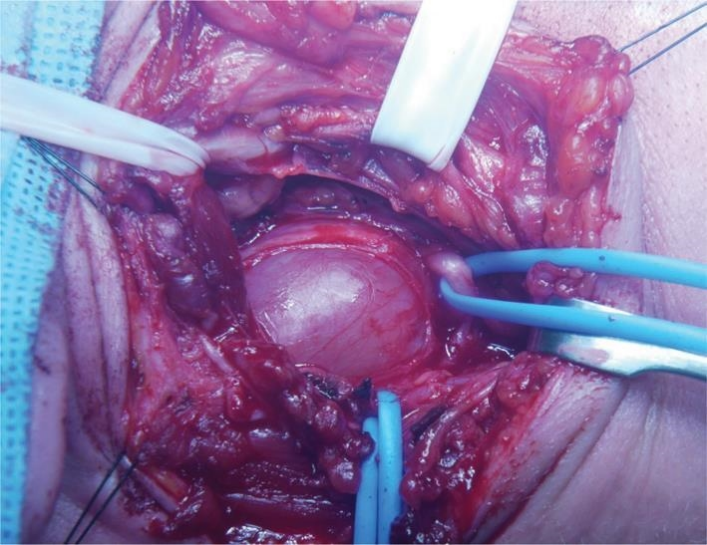
Surgical exploration showing capsular tumor, suggesting schwannoma. Blue vessel loop—vagal nerve.

**Figure 5:**
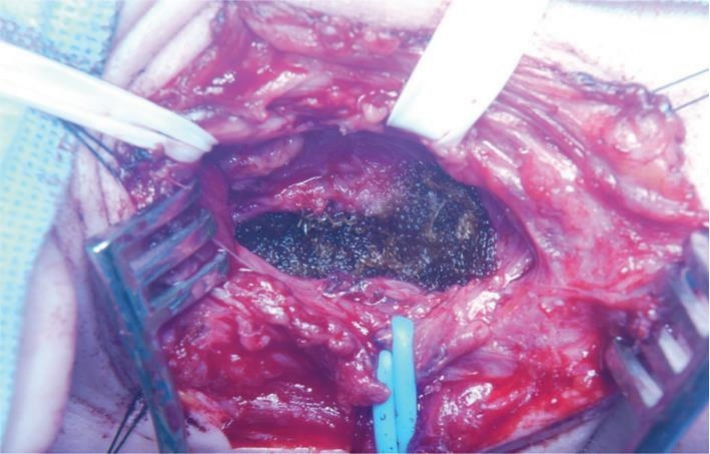
Surgical exploration showing complete resection of tumor. Blue vessel loop—vagal nerve preservation.

**Figure 6:**
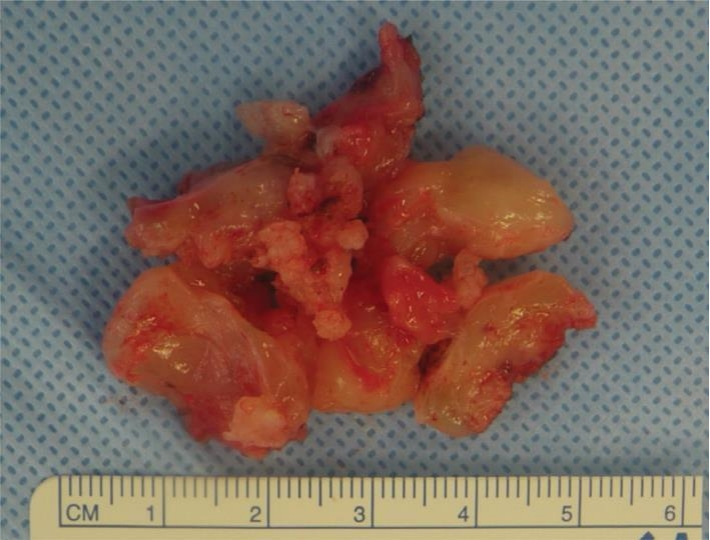
Macroscopic aspect of the tumor.

Surgery was carried out 2 months after diagnosis through a high transverse cervical incision in the posterior triangle of the neck (transcervical approach). During access to the carotid space, a wine-colored mass with a fibroelastic consistency measuring around 4 cm × 3.5 cm was identified (Figure 4). Proximal and distal dissection made it possible to establish the origin of the lesion in the vagus nerve (Figure 5). An epineurotomy was performed in the most superficial area of the tumor, in which no fascicles were identified. Low-intensity electrical stimulation did not result in any muscle contractions or electrocardiographic changes in this area. The tumor was progressively isolated after opening its pseudocapsule longitudinally until the plane of the true capsule was identified, which allowed circumferential dissection of the tumor and identification of the origin and exit fascicles. These fascicles were sectioned to allow complete intracapsular enucleation of the tumor (intracapsular excision) (Figure 6). Viable fascicles are displaced posteriorly and preserved.

## 4. Outcome and Follow-up

Histological examination confirmed the diagnosis of benign schwannoma. No symptoms that might suggest nerve involvement, such as nausea, vomiting, heart rhythm symptoms, altered swallowing, or hoarseness, were observed. However, after surgery, the patient had paresis related to the depressor muscle of the angle of the mouth and depressor of the lower lip, probably due to damage to the mandibular branch of the facial nerve as a result of the separation. He also experienced paresthesia in the lower part of the ear pinna. Three months after surgery, the symptoms resolved completely, and 12 months after surgery, the patient was asymptomatic.

## 5. Discussion

Schwannomas of the cervical portion of the vagus nerve are rare, and their incidence in the general population varies according to the origin of the clinical series, which is reduced in series that include tumors distributed throughout the body and increased in series limited to neck tumors. In a series of 234 cases of solitary schwannoma that underwent surgery, no vagus nerve schwannoma was diagnosed [1], while in the series by Guha et al. [11] with 201 benign and malignant neural sheath tumors, only one case of vagus nerve schwannoma was reported (0.4%). In 442 cases of benign neural sheath tumors of the cervical region and extremities, the incidence of vagus nerve schwannoma was 0.9% [12]. Considering only extracranial schwannomas of the head and neck, Leu and Chang [13] reported 2 out of a total of 52 patients (3.8%).

The incidence of schwannomas in the cervical region is already low; however, when considering nonvestibular schwannomas, the incidence is even lower. Therefore, the presence of these lesions can pose a diagnostic challenge, as differential diagnoses include paraganglioma, branchial cleft cyst, inflammatory adenopathies, malignant lymphoma, metastatic cervical lymphadenopathies, submandibular salivary gland tumors, carotid artery aneurysm, lipoma, hemangioma, and dermoid cyst [14–16].

In this case, we believe that surgery is the most prudent measure because of the risk of ischemic stroke due to compression of the common carotid and vertebral arteries. Vertebral artery stroke is associated with a high mortality rate and a high risk of neurological sequelae.

In this case, we considered surgery as the best therapeutic option for the patient for three reasons:

1. To achieve a definitive histological diagnosis, given that the patient did not present a typical clinical picture where the main symptom is dysphonia.
2. There is a high risk of acute ischemic events with potential fatalities (especially from vertebral artery occlusion) or neurological sequelae.
3. As previously mentioned, the incidence of malignant transformation of these lesions is not negligible, potentially reaching 13%. Therefore, resection of the lesion is the best option to prevent malignant transformation, particularly since the patient is young and has a life expectancy of many years.

The present case brings up a very interesting discussion about whether to perform asymptomatic and carotid and vertebral compressions. To the best of our knowledge, this is the first case report with compression of both arteries leading to an imminent risk of mortality.

In this case, the authors intend to elucidate that tumors in the cervical region should always be investigated with imaging examinations with vessel studies; in case of obvious compression, the best measure is to operate while asymptomatic, especially if the anterior and posterior circulation is compromised.

In a series that included peripheral nerve schwannomas distributed throughout the body, Sandler et al. [10] reported that, until 2019, only 235 cases had been reported.

## Data Availability

Data sharing is not applicable to this article as no new data were created or analyzed in this study.
